# Co-Exposure to Food-Grade and Nano-TiO_2_ with High-Fat Diet Induces Multi-Organ Injury in Liver, Intestine, Brain, and Testicles

**DOI:** 10.3390/toxics14040350

**Published:** 2026-04-21

**Authors:** Ying Ma, Nairui Yu, Yi Zhang, Jiaqi Shi, Xinyan Zhou, Xiaojin Li, Li Guan, Guang Jia, Zhangjian Chen

**Affiliations:** 1 Department of Occupational and Environmental Health Sciences, School of Public Health, Peking University, Beijing 100191, China; mayingmmyy@163.com (Y.M.); 1810306112@bjmu.edu.cn (N.Y.); 1710306142@pku.edu.cn (Y.Z.); 18811050850@163.com (J.S.); 1910306226@pku.edu.cn (X.Z.); 2010306128@stu.pku.edu.cn (X.L.); 13691101800@163.com (L.G.); jiaguangjia@bjmu.edu.cn (G.J.); 2 Beijing Key Laboratory of Toxicological Research and Risk Assessment for Food Safety, School of Public Health, Peking University, Beijing 100083, China

**Keywords:** titanium dioxide nanoparticles, E171, high-fat diet, multi-organ toxicity, neurotoxicity, reproductive toxicity

## Abstract

Titanium dioxide nanoparticles (TiO_2_ NPs), widely used as food additives, frequently coexist with high-fat diets (HD) in modern dietary patterns, yet their combined in vivo toxicity remains poorly understood. This study investigated the multi-organ effects of co-exposure to TiO_2_ NPs or food-grade E171 and HD in male C57BL/6J mice. Mice were randomly assigned to six groups and fed regular or high-fat diets containing 1 wt% TiO_2_ NPs or E171 for 13 weeks. Histopathology, serum biochemistry, organ coefficients, and open-field behavioral tests were used to assess tissue injury and functional alterations. Co-exposure to TiO_2_ NPs and HD markedly exacerbated tissue damage across multiple organs. In the liver, more severe ballooning degeneration, necrosis, and inflammatory infiltration were observed, accompanied by altered liver enzymes and reduced organ coefficients. Intestinal injury was characterized by crypt distortion and increased inflammation, particularly in the HD + TiO_2_ group. Testicular tissues showed disorganized seminiferous tubules, loss of spermatogenic cells, and interstitial hyperplasia. In the brain, hippocampal neurons exhibited pyknosis and disarray, with decreased brain coefficients and impaired exploratory behavior. E171 induced similar but milder effects. These findings indicate that HD enhances TiO_2_ NPs induced multi-organ toxicity, highlighting the health risks of realistic co-exposure to dietary nanoparticles and high-fat foods.

## 1. Introduction

The rapid development of nanotechnology has led to titanium dioxide nanoparticles (TiO_2_ NPs) becoming one of the most widely produced engineered nanomaterials, with global production exceeding 7.8 million tons per year [1]. Due to their excellent whitening properties, TiO_2_ NPs are extensively used in food additives and cosmetics [2,3]. In the food industry, they are commonly used as a pigment under the label E171, primarily to enhance brightness and opacity in products such as candies, chewing gums, powdered sugar coatings, and creamy desserts. Food-grade E171 typically consists of a mixture of micro- and nano-sized TiO_2_ particles, with the microparticle fraction generally exceeding 100 nm and extending into the submicron to low-micrometer range, while up to 36% of the particles fall within the nanoscale range (<100 nm). This nanoscale fraction is of toxicological concern, as it may increase the potential for absorption, bioaccumulation, and biological reactivity compared with bulk TiO_2_. TiO_2_ NPs are structurally similar to the nanoscale fraction of E171 but are more uniform in size and surface activity. Previous studies have reported that TiO_2_ NPs can induce oxidative stress, inflammation, and metabolic dysfunction across multiple organs, including the liver, gut, and reproductive system. Few studies have systematically compared the effects of food-grade E171 and TiO_2_ NPs under the same exposure conditions.

A high-fat diet (HD) is another critical environmental factor that alters systemic metabolism and increases susceptibility to xenobiotic toxicity. HD consumption can induce hepatic steatosis, insulin resistance, and intestinal barrier dysfunction, thereby promoting oxidative stress and inflammation in metabolic organs such as the liver and colon [4,5,6]. Moreover, E171 and TiO_2_ NPs are frequently ingested together with high-fat foods such as chocolates, cream fillings, and baked products, suggesting a realistic co-exposure scenario. Therefore, the potential for synergistic toxicity between TiO_2_-based materials and high-fat dietary conditions warrants detailed investigation.

Recent studies have drawn increasing attention to the interaction between TiO_2_ NPs and dietary components, especially high-fat diets (HD), which are themselves a major contributor to metabolic disorders such as obesity, metabolic dysfunction-associated fatty liver disease (MAFLD), and type 2 diabetes [7,8,9]. Parallel research has demonstrated that TiO_2_ NPs disrupt metabolic pathways via reactive oxygen species (ROS), mitochondrial damage, inflammation, and metabolic dysregulation in liver and gut tissues. An increasing amount of data indicates that co-exposure situations, which more closely resemble actual human exposure patterns, should be taken into account when assessing the biological effects of nanomaterials rather than evaluating them separately [10,11]. Notably, high-fat diets may modify the biodistribution, cellular uptake, and toxicity profile of nanoparticles, while nanoparticles themselves may exacerbate HD-induced metabolic disturbances and inflammatory responses [12]. However, the combined toxicological effects of TiO_2_ NPs or E171 with HD on multiple metabolic and reproductive organs remain largely unexplored.

In this study, we aimed to compare the systemic effects of food-grade E171 and TiO_2_ NPs in C57BL/6J mice under both regular-diet (RD) and high-fat diet (HD) conditions. The exposures were designed to mimic realistic oral ingestion scenarios, with materials mixed into food pellets at 1 wt% for 13 weeks. We hypothesized that the combination of TiO_2_-based materials and HD would induce synergistic multi-organ toxicity, particularly in metabolic and reproductive systems. Histological and biochemical analyses were conducted in the liver, colon, brain, and testicles to elucidate organ-specific responses and potential interactive mechanisms.

## 2. Materials and Methods

### 2.1. Materials and Characterization

Titanium dioxide nanoparticles (TiO_2_ NPs, anatase form, average particle size ~25 nm, ≥99.8% purity) were purchased from Macklin Biochemical Co., Ltd. (Shanghai, China). Food-grade titanium dioxide (E171) was obtained from Shanghai Jianghu Titanium Dioxide Chemical Products Co., Ltd. TiO_2_ NPs and E171 were respectively mixed with regular diet or high-fat diet at a concentration of 1 wt% using a commercial food mixer (Jiangsu Xietong Pharmaceutical Bio-Engineering Co., Ltd. Yangzhou, China) to ensure uniform distribution. The regular diet (RD) contained ~12% kcal from fat, while the high-fat diet (HD) provided 60% kcal from fat.

### 2.2. Animal Experiment

Male C57BL/6J mice (6 weeks old, 18–22 g) were obtained from the Department of Experimental Animal Science, Peking University Health Science Center, and housed under specific pathogen-free (SPF) conditions (22 ± 2 °C, 55 ± 10% humidity, 12 h light/dark cycle) with free access to food and water. After one week of acclimation, mice were randomly divided into six groups (*n* = 8 per group) and exposed to different diets for 13 consecutive weeks. The groups included: a control group receiving regular diet (RD), a group fed regular diet supplemented with 1% food-grade titanium dioxide (E171), and a group receiving regular diet containing 1% titanium dioxide nanoparticles (TiO_2_ NPs); similarly, another three groups were fed a high-fat diet (HD) alone, HD supplemented with 1% E171, or HD with 1% TiO_2_ NPs, respectively. All dietary formulations were prepared using a commercial food mixer to ensure uniform mixing of the particles with the feed. In the high-fat diet, 60% of the total calories come from fat. The main sources of lipids are lard and soybean oil, with lard being the primary source of fat, indicating that the lipid portion is mainly composed of triglycerides. The macronutrient distribution of the high-fat diet is that 20% of the calories come from protein, 60% from fat, and 20% from carbohydrates. The detailed dietary components are listed in Table S1 (Supplementary Materials). The 1% E171 and TiO_2_ supplementation level was selected based on the maximum concentration permitted by the U.S. Food and Drug Administration (FDA) for titanium dioxide used as a food color additive, which is not more than 1% by weight of the food (21 CFR 73.575). The 13-week exposure period was selected to model subchronic, long-term dietary exposure, which more closely reflects realistic human intake scenarios. All procedures were performed in accordance with the NIH Guide for the Care and Use of Laboratory Animals, 8th edition and are reported per the Animal Research: Reporting of In Vivo Experiments (ARRIVE) guidelines. The study was approved by the Institutional Review Committee of Peking University (ethics approval number: BCJB0068, approval date: 20 February 2023).

The experimental duration was 13 weeks, which was chosen to mimic chronic dietary exposure and to allow for sufficient time for metabolic alterations to develop. Every three days, new food was supplied, and both the fresh and surplus diets were measured. Although the precise ingested nanoparticle dose could not be quantified due to feeding variability, all groups had comparable food intake (no statistical difference), ensuring consistent exposure. At the end of the treatment period, mice were anesthetized. Blood samples were collected, and major organs including the liver, intestine, brain, and testicles were harvested for further analysis.

### 2.3. Serum Biochemical Analysis

Blood samples were allowed to clot at room temperature for 30 min and then centrifuged at 3000 rpm for 10 min at 4 °C. The collected serum was stored at −80 °C until analysis. Serum levels of alanine aminotransferase (ALT), aspartate aminotransferase (AST), and alkaline phosphatase (ALP) were determined using an automated biochemical analyzer.

### 2.4. Histological Examination

The liver, colon, brain, and testicles tissues were fixed in 4% paraformaldehyde for 24 h, embedded in paraffin, and cut into 5 µm sections. After deparaffinization and rehydration, the tissue sections were stained with hematoxylin and eosin (H&E) according to standard histological procedures. Stained slides were examined using a light microscope, and representative images were captured at 200× magnifications.

### 2.5. Open Field Test

To evaluate exploratory behavior and anxiety-like responses, an open field test (OFT) was conducted during the final week of exposure, as previously described. Each mouse was placed individually in the center of a square open field arena (50 cm × 50 cm × 40 cm, with black walls and a white floor) under low ambient lighting. The test duration was 5 min per mouse. A digital tracking system was used to record the movement trajectory and analyze behavioral parameters. The arena was virtually divided into central and peripheral zones. The percentage of time spent in the center zone (center time ratio) was calculated to assess anxiety-related behavior. The apparatus was cleaned with 75% ethanol between trials to eliminate olfactory cues.

### 2.6. Statistical Analysis

All data were presented as mean ± standard deviation (SD). Statistical analyses were performed using GraphPad Prism 9.0 (GraphPad Software, San Diego, CA, USA). Differences among groups were evaluated using one-way analysis of variance (ANOVA) or Student’s *t* test. A *p*-value < 0.05 was considered significant.

## 3. Results

### 3.1. Physicochemical Characterization of TiO_2_

**Figure 1:**
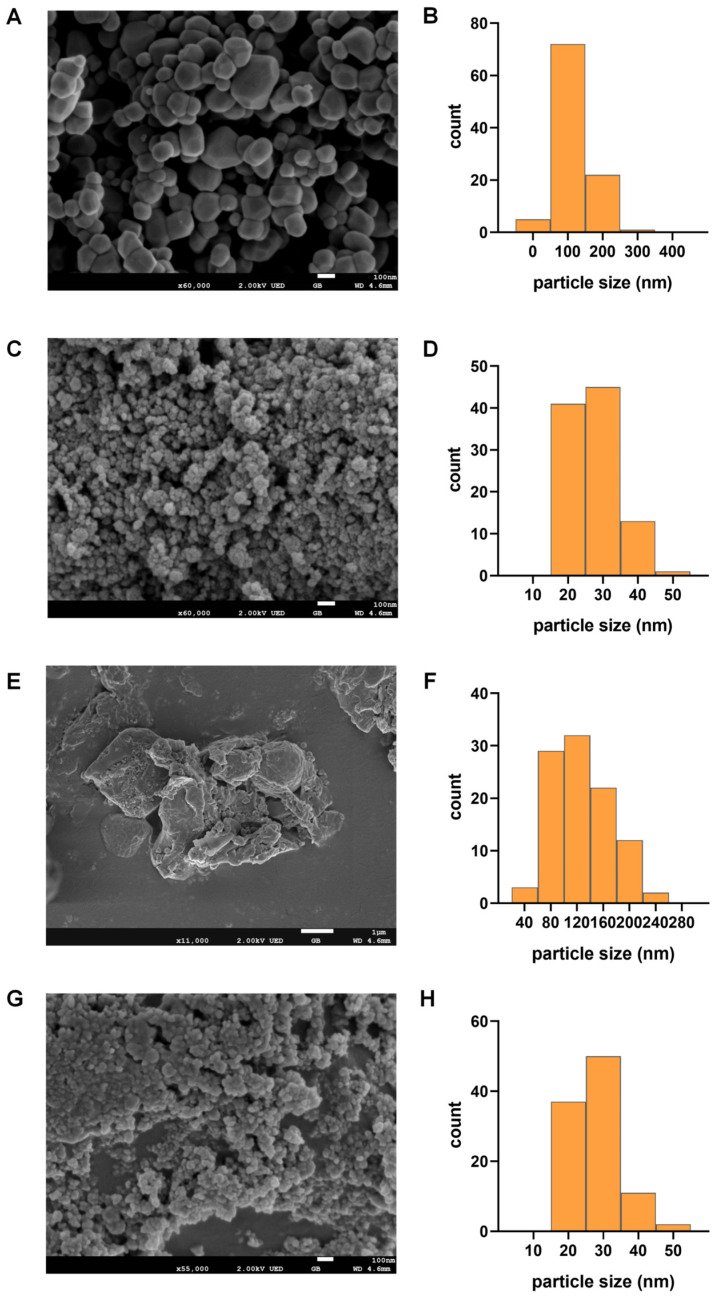
Morphology and particle size distribution of E171 and TiO_2_ NPs. (**A**,**C**,**E**,**G**) Representative SEM images of food-grade TiO_2_ (E171) and TiO_2_ NPs. (**B**,**D**,**F**,**H**) Particle size distribution of E171 and TiO_2_ NPs. Scale bar = 100 nm (**A**,**C**,**G**), Scale bar = 1 μm (**E**).

Both TiO_2_ NPs and E171 used in this study were anatase-type TiO_2_ with a near-spherical morphology, as confirmed by scanning electron microscopy (SEM). The average primary particle size of E171 was 116.51 ± 49.14 nm, whereas that of TiO_2_ NPs was 27.28 ± 6.77 nm (Figure 1). After mixing with regular or high-fat diet using a commercial blender, no significant morphological changes were observed. The post-mixing average particle sizes were 126.38 ± 42.16 nm for E171 and 27.37 ± 6.72 nm for TiO_2_ NPs, respectively. These results suggest that both materials remained relatively stable in size and shape under dietary exposure conditions.

### 3.2. Liver Injury Is Aggravated by Combined Exposure

**Figure 2:**
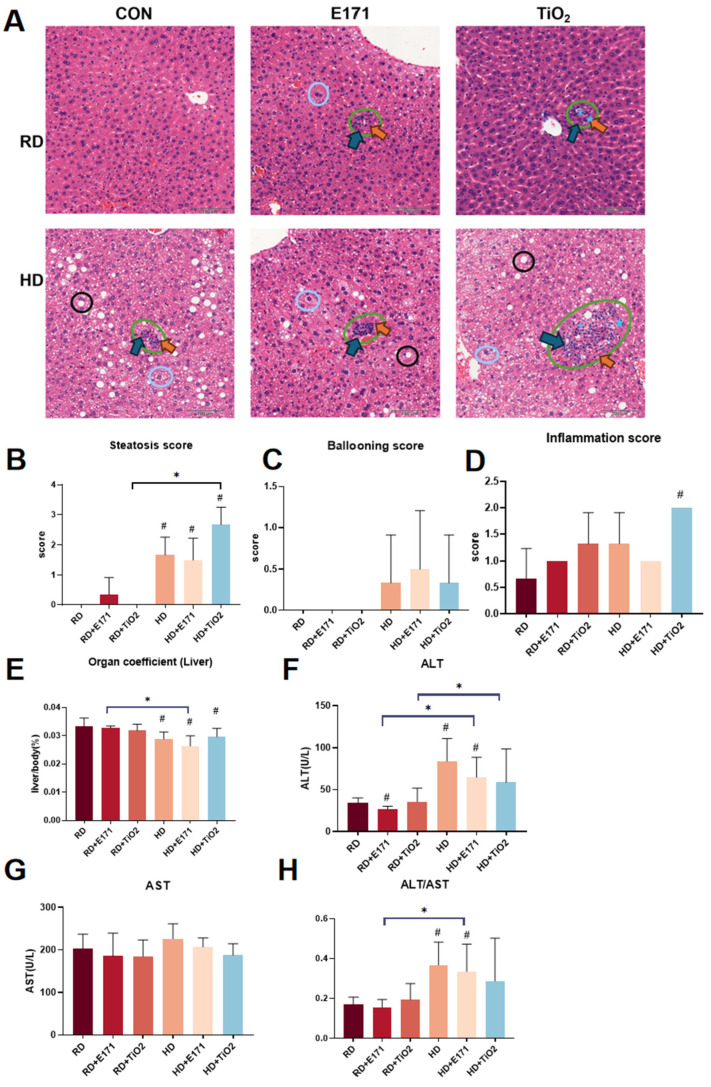
Hepatic histopathological and biochemical changes induced by High-Fat Diet and TiO_2_ NPs exposure. (**A**) Representative H&E-stained liver sections from each group. Blue circles indicate hepatocyte ballooning; green circles denote necrotic foci; black circles highlight lipid droplet accumulation (steatosis); green arrows indicate macrophage infiltration; yellow arrows indicate inflammatory cell infiltration; five-pointed stars indicate apoptotic bodies. Scale bar = 100 μm (**B**) Steatosis score: <5%, 0 point; 6–33%, 1 point; 34–66%, 2 points; >66%, 3 points. (**C**) Ballooning score: none, 0 point; few ballooned hepatocytes, 1 point; many or prominent ballooned hepatocytes, 2 points. (**D**) Inflammation score (20× field): none, 0 point; <2 foci, 1 point; 2–4 foci, 2 points. (**E**) Liver organ coefficients (liver weight/body weight × 100%). (**F**–**H**) Serum levels of ALT (**F**), AST (**G**), and the ALT/AST ratio (**H**). Data are expressed as mean ± SD. # *p* < 0.05 vs. RD; * *p* < 0.05. Group abbreviations: RD: Regular diet group. HD: High-fat diet group. E171: Groups receiving 1% (*w*/*w*) food-grade TiO_2_ (E171) mixed into RD or HD. TiO_2_: Groups receiving 1% (*w*/*w*) titanium dioxide nanoparticles (TiO_2_ NPs) mixed into RD or HD. CON: Control groups fed with RD or HD only, without TiO_2_ additives.

Histological analysis showed that the liver structure in the RD was normal, while the RD + TiO_2_ NP and RD + E171 groups exhibited mild infiltration of inflammatory cells in local areas, suggesting mild hepatic immune activation even under non-obesogenic conditions (Figure 2A). In the high-fat diet (HD) group, hepatocytes displayed marked cytoplasmic vacuolization and ballooning degeneration, indicative of steatosis and early lipotoxic injury. Notably, co-exposure to TiO_2_ with HD significantly aggravated hepatic damage. Histological features included the presence of apoptotic bodies, focal areas of hepatocellular necrosis, and prominent macrophage aggregation around necrotic zones. Inflammatory cell infiltration was more extensive. Both the HD + E171 and HD + TiO_2_ NP groups showed significantly worse hepatic pathology compared to their respective single exposure counterparts (RD + E171 and RD + TiO_2_ NPs) (Figure 2B–D).

The liver organ coefficient significantly decreased in the HD, HD + E171, and HD + TiO_2_ groups (*p* < 0.05) (Figure 2E). Furthermore, the HD + E171 group exhibited a significantly lower liver coefficient than the RD + E171 group (*p* < 0.05), indicating that high-fat conditions may reduce relative liver mass. Serum biochemical analysis further supported these findings (Figure 2F–H). ALT levels were significantly decreased in the RD + E171 group compared to RD (*p* < 0.05), while both the HD and HD + E171 groups showed significantly increased levels (*p* < 0.05). Moreover, ALT levels in the HD + E171 and HD + TiO_2_ groups were significantly higher than those in the RD + E171 and RD + TiO_2_ groups (*p* < 0.05). The results indicate that the combination of TiO_2_ (or E171) with HD exceeded the toxicity when exposed alone.

### 3.3. Intestine Injury and Inflammation

**Figure 3:**
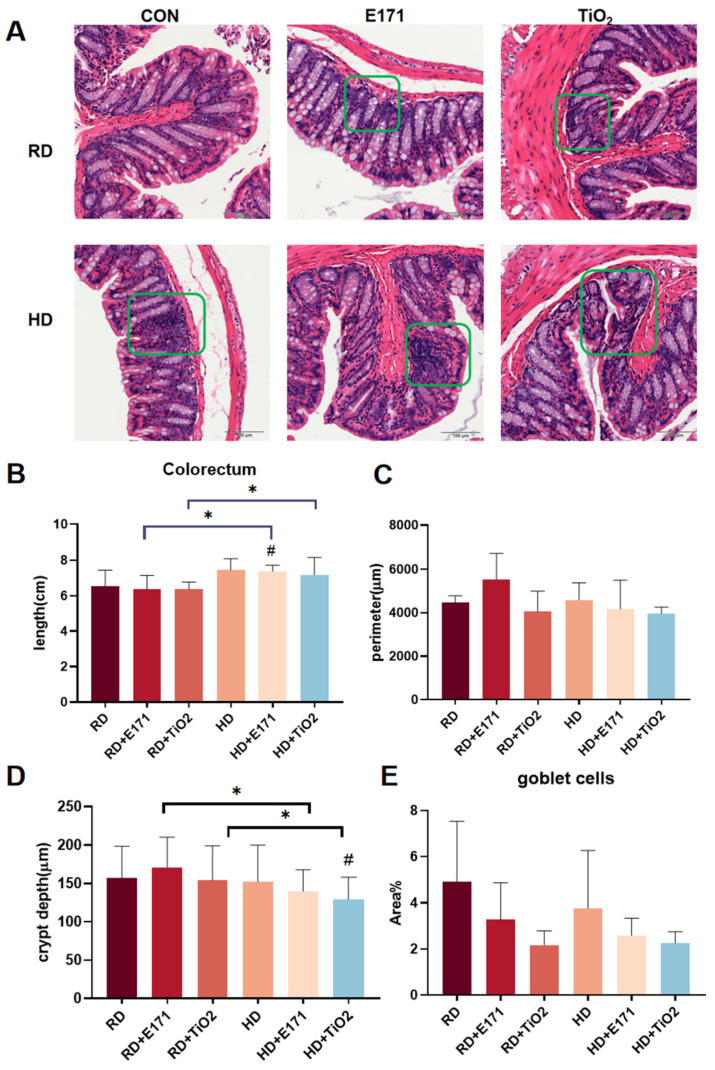
Intestinal histopathological changes induced by High-Fat Diet and TiO_2_ NP exposure. (**A**) Representative H&E-stained sections of colon from each group. Green boxes indicate regions of inflammatory cell infiltration. Scale bar = 100 μm. (**B**) Measured colorectal length (from cecum to anus). (**C**) Cross-sectional circumference of the colon lumen. (**D**) Crypt depth. (**E**) Goblet cell area ratio. Data are presented as mean ± SD. # *p* < 0.05 vs. RD; * *p* < 0.05, compared to other groups. Group abbreviations: RD: Regular diet group. HD: High-fat diet group. E171: Groups receiving 1% (*w*/*w*) food-grade TiO_2_ (E171) mixed into RD or HD. TiO_2_: Groups receiving 1% (*w*/*w*) titanium dioxide nanoparticles (TiO_2_ NPs) mixed into RD or HD. CON: Control groups fed with RD or HD only, without TiO_2_ additives.

Histological assessment of the colon in the RD group revealed intact epithelial lining, well-preserved crypt architecture, and normal mucosal thickness. In contrast, mice in the RD + TiO_2_ and RD + E171 groups exhibited mild distortion of crypt structure along with limited inflammatory cell infiltration in the lamina propria, indicating that adding nano/micron materials to the diet can stimulate the colon (Figure 3A). However, co-exposure with HD markedly aggravated these pathological changes compared with single exposures. The intestinal structure was more disrupted, with increased inflammatory cell infiltration and crypt atrophy observed under combined treatment.

The colorectum length further supported these histological observations (Figure 3B). Compared with TiO_2_ or E171 exposure alone, co-exposure with HD significantly increased colorectal length, which may reflect a compensatory or pathophysiological intestinal response [13]. Morphometric analysis showed that crypt depth was significantly reduced and the goblet cell area ratio decreased in co-exposed groups compared with single-exposure groups, indicating exacerbated crypt atrophy and impaired mucosal barrier integrity (Figure 3D,E).

### 3.4. Testicles: Co-Exposure Leads to Spermatogenic Failure and Sertoli Cell-Only Syndrome

**Figure 4:**
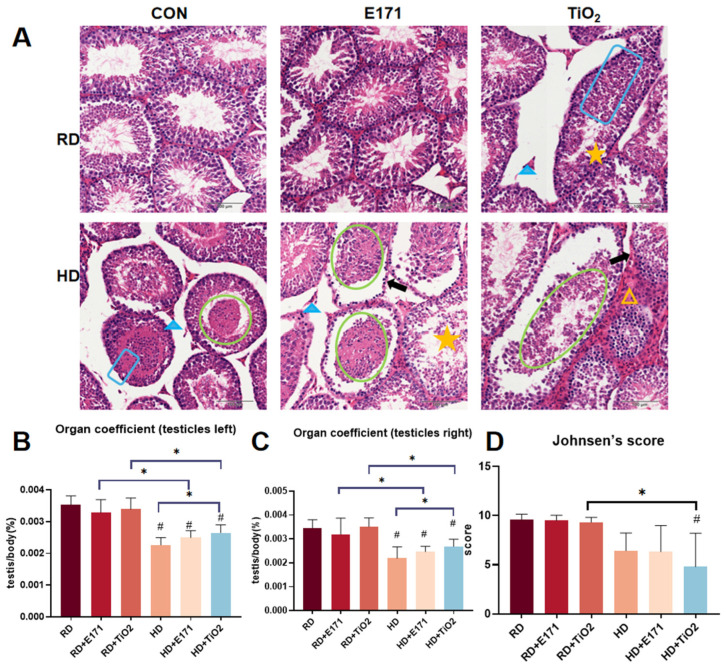
Testicular pathological changes induced by High-Fat Diet and TiO_2_ NPs exposure. (**A**) Representative H&E-stained testicles sections from each group. Blue boxes indicate seminiferous tubules; green circles indicate exfoliation of spermatogenic cells; blue triangles indicate degeneration of interstitial cells; yellow triangles indicate interstitial cell hyperplasia; black arrows indicate thickening of the basement membranes; stars indicate impaired spermatogenesis. Scale bar = 100 μm. (**B**,**C**) Organ coefficients of the left (**B**) and right (**C**) testicles (testicles weight/body weight × 100%). (**D**) Johnsen’s score (0–10) was calculated by averaging the per-animal score. Data are presented as mean ± SD. # *p* < 0.05 vs. RD; * *p* < 0.05, compared to other groups. Group abbreviations: RD: Regular diet group. HD: High-fat diet group. E171: Groups receiving 1% (*w*/*w*) food-grade TiO_2_ (E171) mixed into RD or HD. TiO_2_: Groups receiving 1% (*w*/*w*) titanium dioxide nanoparticles (TiO_2_ NPs) mixed into RD or HD. CON: Control groups fed with RD or HD only, without TiO_2_ additives.

Histological analysis showed that the RD group and the RD + E171 group had normal testicular architecture, including well-organized seminiferous tubules, intact basement membranes, and healthy interstitial Leydig cells (Figure 4A). However, RD + TiO_2_ resulted in visible disruption of the seminiferous tubule structure. The tubules showed disorganized germinal layers, reduced spermatogenesis, and degenerative changes in the interstitial cells, indicating early signs of testicular toxicity even in the absence of a high-fat diet. In the HD group, testicular tissues displayed evident degeneration, with exfoliation of germ cells into the lumen of seminiferous tubules, disorganized cellular arrangement, and progressive Leydig cell atrophy. These effects were more pronounced in the combined exposure group. The seminiferous tubules exhibited extensive germ cell exfoliation accompanied by thickening of the basement membrane and reduced spermatogenic activity. These lesions were further characterized by prominent interstitial cell hyperplasia and features consistent with Sertoli cell-only syndrome, indicating severe disruption of spermatogenesis and testicular architecture.

Testicular organ coefficients (testicles weight/body weight × 100%) decreased in the HD, HD + E171, and HD + TiO_2_ groups (*p* < 0.05) (Figure 4B,C). Both the left and right testicles showed similar trends. Interestingly, the HD + E171 and HD + TiO_2_ groups exhibited significantly higher testicular coefficients compared to their respective RD + E171 and RD + TiO_2_ counterparts (*p* < 0.05), potentially reflecting compensatory hypertrophy. Moreover, the testicular coefficient of the HD + TiO_2_ group was significantly increased relative to the HD group (*p* < 0.05), indicating possible nanoparticle-induced modulation of testicular mass under lipid-stressed conditions. Johnsen’s score analysis revealed a significant decrease in the HD + TiO_2_ group compared to the RD and RD + TiO_2_ groups (*p* < 0.05) (Figure 4D). This suggests that co-exposure significantly increased testicular toxicity.

### 3.5. Brain Histopathological and Functional Changes Induced by Co-Exposure

**Figure 5:**
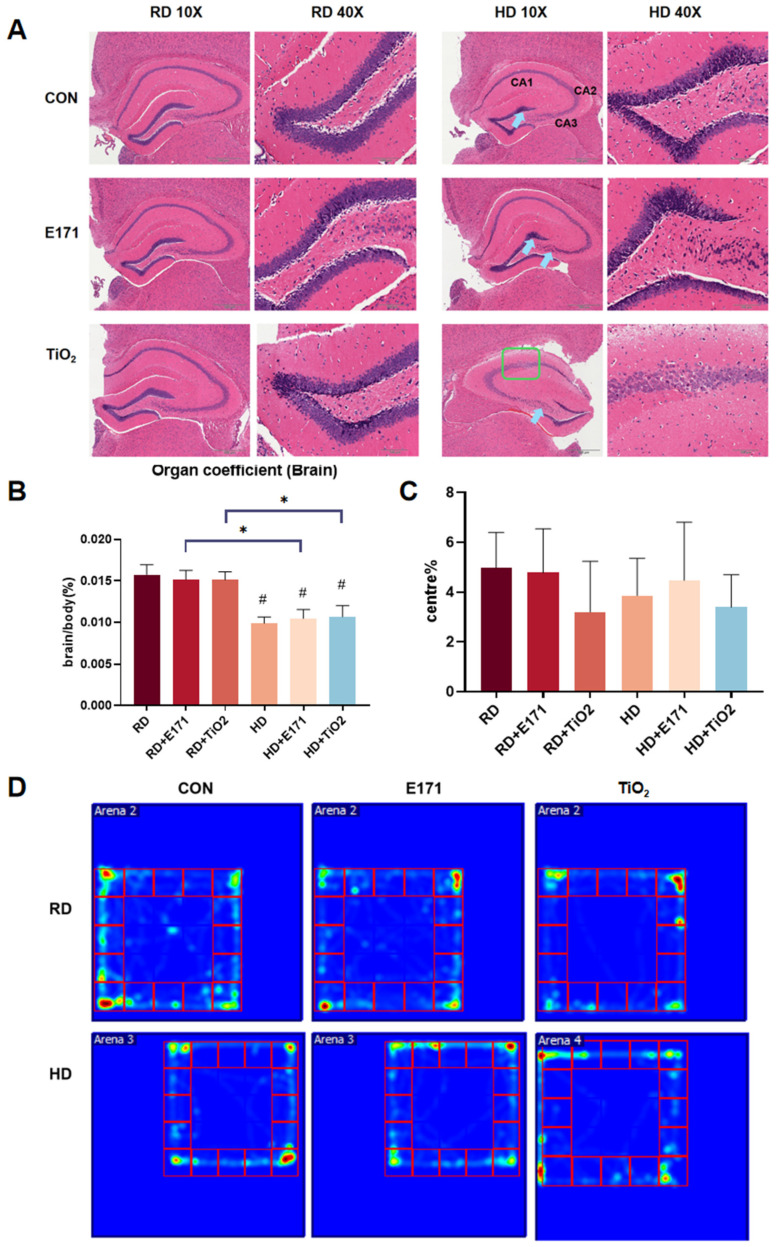
Effects of High-Fat Diet and TiO_2_ NPs on hippocampal structure and exploratory behavior. (**A**) Representative H&E-stained hippocampal sections from each group. Blue arrows indicate neuronal nuclear pyknosis; green boxes highlight areas of disorganized or loose neuronal arrangement. Scale bar = 100 μm or 500 μm. (**B**) Brain organ coefficients (brain weight/body weight × 100%). (**C**) Center zone entry ratio in the open field test. (**D**) Heatmap of movement trajectories in the open field test across different groups. Data are presented as mean ± SD. # *p* < 0.05 vs. RD; * *p* < 0.05, compared to other groups. Group abbreviations: RD: Regular diet group. HD: High-fat diet group. E171: Groups receiving 1% (*w*/*w*) food-grade TiO_2_ (E171) mixed into RD or HD. TiO_2_: Groups receiving 1% (*w*/*w*) titanium dioxide nanoparticles (TiO_2_ NPs) mixed into RD or HD. CON: Control groups fed with RD or HD only, without TiO_2_ additives.

Histological examination of the hippocampal region—an area critically involved in cognition and emotional regulation—revealed normal architecture in the RD, RD + E171, and RD + TiO_2_ groups (Figure 5A). In these mice, hippocampal neurons displayed regular morphology, tightly packed arrangement, large round nuclei, and clear nucleoli. In contrast, mice in the co-exposure groups exhibited pathological alterations consistent with neurotoxicity. In these groups, some hippocampal neurons demonstrated signs of cellular degeneration, including nuclear pyknosis and cytoplasmic shrinkage. Notably, the HD + TiO_2_ group presented with the most pronounced alterations—neuronal arrangement in the hippocampal CA1 and CA3 regions appeared loose and disorganized, suggesting progressive structural disruption of the hippocampal network.

The brain organ coefficient (brain weight/body weight × 100%) was significantly decreased in the HD, HD + E171, and HD + TiO_2_ groups compared to the RD group (*p* < 0.05), indicating potential neurodevelopmental or neurodegenerative effects associated with lipid overload and particle exposure (Figure 5B). Furthermore, the HD + E171 and HD + TiO_2_ groups showed substantially lower brain coefficients compared to single exposure (*p* < 0.05), implying that combined exposure had adverse impacts on brain.

To preliminarily assess behavioral implications of these structural changes, an open field test was performed to examine anxiety-like behavior and locomotor activity. While total movement did not differ substantially between groups, a slight reduction in center rate was observed in combined exposure groups compared to their single exposure, suggesting a potential increase in anxiety-like behavior or altered exploratory behavior, though these differences did not reach statistical significance (Figure 5C,D). Overall, these findings indicate that co-exposure can lead to structural impairment in the hippocampus and may be associated with subtle neurobehavioral changes.

## 4. Discussion

This study demonstrated that combined exposure to titanium dioxide (E171 or TiO_2_ NPs) and a high-fat diet (HD) induced more severe multi-organ damage than either factor alone. Histopathological and biochemical analyses consistently revealed that co-exposure exacerbated hepatic steatosis, intestinal barrier disruption, testicular degeneration, and hippocampal neuronal injury. These data highlight that real-world dietary exposure conditions, in which nanoparticles and fatty foods commonly mix, may have underestimated health risks.

Due to its central role in detoxification, lipid metabolism, and immune surveillance, the liver is particularly susceptible to damage induced by both metabolic stress and engineered nanomaterials. Previous studies have shown that TiO_2_ NPs can induce hepatic oxidative stress, inflammation, and lipid dysregulation [14,15]. High-fat diets can further compound these effects by promoting hepatic steatosis, insulin resistance, and mitochondrial dysfunction [16,17]. In our study, mice co-exposed to HD and TiO_2_ (or E171) exhibited greater ballooning degeneration and inflammatory infiltration compared to those with single exposures. In our previous meta-analysis, it was found that exposure to TiO_2_ NPs was significantly associated with elevated levels of ALT and AST [14]. In this study, ALT and ALT/AST ratios were changed, supporting the presence of hepatocellular damage. Although conventional serum markers of liver injury were assessed in this study, γ-glutamyl transferase (GGT) was not included, which may limit the evaluation of bile ductular function and potential cholestatic alterations. Future studies should incorporate GGT to provide a more comprehensive assessment of hepatobiliary injury associated with TiO_2_ exposure. Moreover, liver organ coefficients were significantly reduced in co-exposure groups, indicating structural or metabolic injury. These findings are consistent with previous reports that TiO_2_ NPs promote oxidative stress and inflammation, all of which exacerbate steatohepatitis under high-fat dietary conditions [15,16].

The intestine functions as the primary interface for orally ingested nanoparticles and plays a crucial role in regulating host–microbiota interactions and systemic immune responses. Previous studies have shown that TiO_2_ NPs can cause local inflammation, change the composition of the gut microbiota, and disrupt intestinal epithelial tight junctions [17,18]. Also, high-fat diets are known to impair intestinal barrier function and promote systemic endotoxemia through increased permeability and chronic low-grade inflammation [19]. Despite this growing concern, comprehensive histopathological evaluations of intestinal tissues under combined exposure to TiO_2_ NPs and HD are still limited. Our results further revealed that co-exposure can aggravate crypt atrophy, mucosal inflammation, and goblet cell loss, reflecting barrier impairment. Interestingly, colorectal length was significantly increased in combined groups. Although shortening is common in acute inflammation, chronic low-grade inflammation may lead to compensatory elongation, which may reflect tissue remodeling or an adaptive response to maintain barrier integrity under chronic stress conditions [13].

Another organ system that deserves mention in this regard is the male reproductive system, specifically the testicles. TiO_2_ NPs have been shown to accumulate in testicular tissue and disrupt spermatogenesis, testosterone production, and sperm motility in animal models [20,21]. Similarly, HD can adversely affect male fertility by inducing testicular oxidative stress, Leydig cell dysfunction, and hormonal imbalances [22,23]. Despite the well-documented reproductive toxicity of both TiO_2_ NPs and HD individually, their combined effects on testicular structure and function remain poorly characterized. In our research, we observed that combined exposure might result in more severe testicular damage than single exposure. There was a significant decrease in the organ coefficient and Johnsen’s score. Mechanistically, oxidative stress appears to play a pivotal role. Ogunsuyi et al. reported that TiO_2_ NP exposure significantly reduced the activities of antioxidant enzymes such as superoxide dismutase (SOD), catalase (CAT), and glutathione (GSH), thereby promoting oxidative damage and structural injury to testicular tissue [24]. In another study, Halawa et al. demonstrated that TiO_2_ NPs not only increased lipid peroxidation in the testicles but also upregulated genes associated with apoptosis and inflammatory pathways [25].

The brain is increasingly recognized as a vulnerable target in the context of nanoparticle-induced toxicity. Although TiO_2_ NPs poorly cross the blood–brain barrier (BBB) under normal conditions, systemic inflammation and lipid-induced endothelial dysfunction can enhance BBB permeability [26]. Once within the brain, TiO_2_ NPs have been shown to trigger neuroinflammatory reactions, glial activation, and oxidative stress, all of which may contribute to progressive neurodegeneration [27,28]. In parallel, high-fat diet has been implicated in the development of neuroinflammation and cognitive dysfunction. The underlying mechanisms include impaired insulin signaling, increased BBB permeability, and dysregulation of the gut–brain axis [29,30]. However, the potential combined effects of TiO_2_ NPs and HD on brain histopathology remain largely unexplored. In our study, we observed that hippocampal neurons maintained normal morphology in RD groups but showed nuclear pyknosis and neuronal disorganization in combined group. These pathological changes are consistent with previous findings reporting glial overactivation, tissue necrosis as well as hippocampal cell apoptosis following chronic TiO_2_ NPs exposure [31]. In the present study, only the hippocampus was examined as a representative brain region because of its well-established sensitivity to oxidative stress, neuroinflammation, and metabolic disturbances. However, other brain regions, including the cortex and prefrontal cortex, may also be affected by TiO_2_ exposure and high-fat diet-related injury. Therefore, the lack of broader brain-region assessment should be considered a limitation, and future studies should include additional regions to provide a more comprehensive evaluation of neurotoxicity. Furthermore, brain organ coefficients were significantly reduced in co-exposure groups, suggesting structural or functional loss. Behavioral data from the open field test revealed a mild reduction in center exploration in co-exposure groups, indicative of potential anxiety-like behavior, although statistical significance was not achieved. This aligns with earlier studies demonstrating that oral TiO_2_ NPs exposure can induce hippocampal oxidative damage, leading to spatial memory impairment and anxiety-like behaviors in rodents [32,33,34].

A limitation of the present study is that histopathological examination was limited to selected organs rather than a full systemic evaluation of all tissues. The intestine was selected as the primary site of oral exposure, the liver as the major metabolic organ involved in high-fat diet-related injury and NAFLD progression, and the brain and testicles as representative extrahepatic organs potentially susceptible to TiO_2_ translocation and secondary oxidative inflammatory damage. Nevertheless, other organs, particularly the kidney, may also be affected by particulate exposure and were not evaluated in the present study. This should be considered a limitation, and future studies should incorporate broader organ histopathology, including the kidney, to provide a more comprehensive systemic toxicological profile.

The enhanced toxicity observed under combined exposure to TiO_2_ (E171 and nanoparticles) and high-fat diets may be attributed to multiple mechanisms. First, HD-induced metabolic stress, such as hyperlipidemia, insulin resistance, and chronic low-grade inflammation, which can compromise organ defense systems and increase tissue susceptibility to exogenous insults [12,35]. These changes facilitate nanoparticle accumulation and potentiate their adverse effects in metabolically active tissues, such as the liver and intestine. For instance, HD can increase intestinal permeability and weaken the mucosal barrier, allowing for greater translocation of ingested nanoparticles into systemic circulation [36,37]. Second, co-exposure can exacerbate oxidative stress and inflammatory responses. Studies have shown that nanoparticles stimulate the production of ROS and activate NF-κB and MAPK signaling pathways, amplifying pro-inflammatory cytokine release [38,39]. In an environment already burdened by inflammation or metabolic stress, such as that induced by a high-fat diet, this can trigger a vicious cycle where tissue injury and inflammation amplify each other. This phenomenon has also been validated in studies involving other nanoparticles combined with high-fat diets. For example, when carbon dots were combined with high-fat diets, glucose homeostasis was disturbed. And co-exposure to lead and high-fat diets can induce hyperglycemia and insulin resistance [40,41].

## 5. Conclusions

In summary, combined exposure to TiO_2_ (or E171) and high-fat diets caused aggravated damage in the liver, intestine, testicles, and brain of mice. These results highlight the significance of reassessing food safety under real-world situations such as the coexistence of high-fat diets and food additives.

## Data Availability

The original contributions presented in this study are included in the article. Further inquiries can be directed to the corresponding author.

## Supplementary Materials

The following supporting information can be downloaded at: https://www.mdpi.com/article/10.3390/toxics14040350/s1, Table S1: Nutritional composition of the high-fat diet.

## Abbreviations

The following abbreviations are used in this manuscript:

ALP	Alkaline phosphatase
ALT	Alanine aminotransferase
AST	Aspartate aminotransferase
BBB	Blood–brain barrier
CAT	Catalase
CON	Control
E171	Food-grade titanium dioxide
GSH	Glutathione
H&E	Hematoxylin and eosin
HD	High-fat diet
MAFLD	Metabolic dysfunction-associated fatty liver disease
MAPK	Mitogen-activated protein kinase
NF-κB	Nuclear factor kappa-B
OFT	Open field test
RD	Regular diet
ROS	Reactive oxygen species
SEM	Scanning electron microscopy
SPF	Specific pathogen-free
TNP	Titanium dioxide nanoparticles
TNF-α	Tumor necrosis factor alpha
