# Supplementary material for: Co-Exposure to Food-Grade and Nano-TiO2 with High-Fat Diet Induces Multi-Organ Injury in Liver, Intestine, Brain, and Testicles
Source: Toxics. 2026 Apr 21;14(4):350. doi: 10.3390/toxics14040350 (PMC13120316; doi:10.3390/toxics14040350)
Supplement: Supplementary file 1 [file toxics-14-00350-s001.zip › toxics-4237816-supplementary.pdf]

Table S1. Nutritional composition of the high-fat diet

Parameter	High-fat diet
Fat-derived energy (%)	60
Protein (kcal%)	20
Fat (kcal%)	60
Carbohydrate (kcal%)	20
Protein (g%)	26
Fat (g%)	35
Carbohydrate (g%)	26
Main fat sources	Lard, soybean oil
Predominant lipid class	Primarily triglycerides
Fiber source	Cellulose
Total energy (kcal/kg)	4037.2
